# Special issue: Genetics of maize–microbe interactions

**DOI:** 10.1111/mpp.13348

**Published:** 2023-05-20

**Authors:** Peter Balint‐Kurti, Guan‐Feng Wang

**Affiliations:** ^1^ USDA‐ARS Plant Science Research Unit Raleigh North Carolina USA; ^2^ Department of Entomology and Plant Pathology North Carolina State University Raleigh North Carolina USA; ^3^ The Key Laboratory of Plant Development and Environmental Adaptation Biology, Ministry of Education, School of Life Sciences, Shandong University Qingdao Shandong China

Maize (*Zea mays*) is an annual grass belonging to the tribe Andropogoneae of the family Gramineae. Its high productivity and adaptability have resulted in it being the world's most produced crop (Erenstein et al., 2022). Global yield loss of maize caused by pathogens and pests was estimated to be 19.5%–41.1% (Savary et al., 2019). In the United States, losses due to diseases were estimated at approximately 7%–10% over recent years (Mueller et al., 2020). The investigation of maize–microbe interactions therefore would seem to be a crucial area of study, and it is the subject of this special issue.

Maize is also a well‐established model genetic system for plant science. Studies on maize have made essential contributions to the elucidation of a number of important concepts, including the nature of transposons, epigenetic inheritance, and heterosis (Strable & Scanlon, 2009). Maize is, in particular, a model system for quantitative genetic studies (Wallace et al., 2014). A number of pioneering studies in the field of plant–microbe interaction have used maize. The first identification of a plant disease resistance gene, *Hm1* (Johal & Briggs, 1992), and of a plant disease susceptibility gene, *T‐urf13* (Dewey et al., 1988), occurred in maize. Studies of the maize *Rp1* locus gave us early indications of the intriguing structure and complexity of some plant disease resistance loci (Saxena & Hooker, 1968; Sudupak et al., 1993). Despite this history, it is probably fair to say that the maize system was not generally in the forefront of molecular plant–microbe research during the late 20th and early 21st century. Perhaps this was due partly to the fact that maize is a big plant, hard to grow in controlled conditions in the greenhouse or growth chamber, and that it is comparatively recalcitrant to genetic transformation, making functional studies challenging. Also, unlike many other crops, maize is generally not directly consumed by humans in the developed world and so important maize diseases have perhaps not received the publicity (and therefore the funding) that diseases of some other crops have enjoyed. Furthermore, many (though not all!) maize diseases have been adequately controlled by genetic resistance incorporated into elite varieties, helped both by the extensive public and private breeding efforts in this crop and by the relatively high genetic diversity available within cultivated maize (Buckler et al., 2006; Yang, Balint‐Kurti, et al., 2017).

In the past 10–15 years there has been an upswell of activity in the study of the genetics of maize–microbe interactions, with respect to both the number of researchers working in the field and the number of important studies that have emerged, some examples of which follow, though it should be noted that this is a subjective and rather incomplete list. The use of powerful mapping populations such as the maize nested association population (Gage et al., 2020) has allowed the detailed description of the genetic architectures controlling quantitative resistance to several important maize diseases and of the defence response (Benson et al., 2015; Kump et al., 2011; Li et al., 2018; Olukolu et al., 2014, 2016; Poland et al., 2011). Several quantitative and major effect resistance genes have been cloned (Chen et al., 2022, 2023; Deng et al., 2022; Hurni et al., 2015; Konlasuk et al., 2015; Leng et al., 2017; Li et al., 2019; Liu et al., 2017; Wang et al., 2022; Yang, He, et al., 2017; Yang et al., 2021; Zuo et al., 2015) and, in at least two cases, the microbial avirulence determinant has also been identified (Chen et al., 2022; Deng et al., 2022). Fascinating work has been reported on the manipulation of maize metabolism by the common smut pathogen *Ustilago maydis* (Redkar et al., 2015; Skibbe et al., 2010; Villajuana‐Bonequi et al., 2019). The molecular mechanisms controlling the activity of the maize nucleotide‐binding, leucine‐rich repeat (NLR) resistance protein Rp1 have been elucidated (Luan et al., 2021; Sun et al., 2023; Wang et al., 2015; Wang & Balint‐Kurti, 2016) and the maize microbiome has been analysed in some detail (Peiffer et al., 2013; Wagner, Busby, et al., 2020; Wagner, Roberts, et al., 2020; Wallace et al., 2018).

In this special issue we demonstrate that this resurgence in interest in the molecular genetics of maize–microbe interactions continues apace. We present papers covering a diverse and representative area of research in the field, including studies on maize‐associated fungi, bacteria, and viruses as well as the maize seed microbiome. These papers cover established and emerging viral tools for functional genomics in maize, a genomic study of maize resistance genes, and the identification and detailed characterization of maize genes involved in pathogen resistance and microbial genes involved in pathogenicity.

The relative difficulty of producing stable transgenic plants in maize is an important disadvantage compared to other model plant systems such as *Arabidopsis*, tomato, and rice. Many functional studies of maize genes have therefore relied on the use of viral systems to transiently express or suppress endogenous gene expression or on the identification of transposon‐induced mutants. In recent years CRISPR/Cas9‐mediated gene editing has also frequently been used for targeted gene modification. This issue includes reports that use all these approaches for functional gene characterization.

Kanakala et al. report the construction of an infectious clone of *Alphanucleorhabdovirus maydis* (also known as maize mosaic virus), making it the first negative‐strand RNA virus vector available for use in maize (2023, this issue). This promises to be an important step both in understanding the biology of this virus and as a tool for functional genomics in maize. As a tool for expression of exogenous genes, this vector offers superior stability and carrying capacity compared to the available alternative systems.

The use of viruses to transiently silence endogenous genes is known as virus‐induced gene silencing (VIGS), and probably the most used VIGS system in maize in recent years has been based on foxtail mosaic virus (FoMV). Beernink and Whitham review how FoMV has been used in maize and other monocots for both gene expression and gene silencing and discuss the advantages and limitations of the system (2023, this issue). Yu et al. used the FoMV system, among other approaches, to identify three maize receptor‐like kinase proteins in the Feronia‐like receptor family (2022, vol. 23, pp. 1331–1345). They report evidence suggesting that these proteins are important in regulating an aspect of basal immunity and affect resistance to multiple diseases.

Zhang et al. report on an integrated analysis of the transcriptome and proteome of the stalks of maize plants infected with *Fusarium verticillioides*, a pathogen that causes both maize stalk and ear rot and produces important mycotoxins (2023, this issue). They identified several pathways that are regulated during infection at both the RNA and the protein level, and using a brome mosaic virus‐based VIGS system they confirm the roles of several candidate genes in resistance. Liao et al., also working with *F*. *verticillioides* but this time on the ear rather than the stalk rot that it causes, report that the maize proteins ZmSIZ1a and ZmSIZ1b possess E3 ligase activity and act redundantly to confer increased resistance to Fusarium ear rot (2023, this issue). In this case they used CRISPR/Cas9 technology to generate mutants for functional studies. Huang et al. investigate the function of the maize oxophytodienoate reductase gene *OPR2*, and using transposon‐induced mutations they show that it has opposite effects with respect to resistance to biotrophs and necrotrophs and that these effects may be associated with the effect of *OPR2* on levels of the plant hormones salicylic acid and jasmonic acid (2023, this issue).

Most major plant disease resistance genes are of the NLR type. Thatcher, Jung et al. (2023, this issue) took advantage of the recent availability of a set of diverse maize genome sequences to characterize the diversity of NLR genes in maize as well as the maize relative *Zea luxurians*. They identified some features of NLRs not noted in the other species examined so far, confirm the relatively low number of NLRs in maize compared to other crop species, and highlight the diversity of NLR sequences and locations among lines and across species. In a separate but related study, Thatcher, Leonard et al. (2023, this issue) describe the map‐based cloning of the *Ht1* resistance gene for northern leaf blight. They find that the encoded protein is an NLR with a coiled‐coil N‐terminal domain, typical of a large class of major plant resistance genes. This is a different structure from the previously reported northern leaf blight resistance gene *Htn1*/*Ht2*/*Ht3*, which encodes a wall‐associated kinase. While *Ht1* confers a high degree of resistance, it is often considered a quantitative resistance gene; it is somewhat unusual for coiled‐coil NLRs to underlie quantitative as opposed to qualitative resistance.

The so‐called “plant–pathogen arms race” and the means used by the pathogen to subvert plant defence mechanisms is an important consideration in any plant pathogen system. Weiland et al. report a detailed structure–function analysis of the chitin‐binding cerato‐platanin protein Cpl1 from the maize pathogen *U. maydis* and its homologue Uvi2 from the barley pathogen *Ustilago hordei*, which may help the pathogen avoid aspects of the plant surveillance system (2023, this issue). They demonstrate that these proteins bind chitin in a previously unreported way and investigate and discuss the similarities and differences in the roles of these proteins in their respective systems.

This issue also includes reviews of two important emerging fields. Osdaghi et al. present a pathogen profile (2023, this issue) of *Clavibacter nebraskensis*, the bacterial agent causing maize Goss's wilt. While this pathogen was discovered in Nebraska in the late 1960s and was an important pathogen in the United States through the 1970s, it was of relatively minor importance subsequently until a resurgence in the mid‐2000s. This timely review discusses the reasons for the disease's re‐emergence and summarizes its biology, taxonomy, and methods for early detection and control. Finally, Wallace reviews our rapidly advancing knowledge of the maize seed microbiome, including what we know about its composition transmission and the interactions with the host and between the microorganisms themselves (2023, this issue).

We feel that this collection of reports together provides a useful snapshot of current research in this area as well as being valuable resources individually.
